# Skin Bacterial and Fungal Microbiome Responses to Diet Supplementation and Rewilding in the Critically Endangered Southern Corroboree Frog

**DOI:** 10.1111/mec.17562

**Published:** 2024-10-21

**Authors:** Alice Risely, Phillip G. Byrne, David A. Hunter, Ana S. Carranco, Bethany J. Hoye, Aimee J. Silla

**Affiliations:** ^1^ School of Science, Engineering and Environment University of Salford Manchester UK; ^2^ Environmental Futures, School of Earth, Atmospheric and Life Sciences University of Wollongong Wollongong New South Wales Australia; ^3^ NSW Department of Climate Change, Energy, the Environment and Water Albury New South Wales Australia; ^4^ Institute of Evolutionary Ecology and Conservation Genomics Ulm University Ulm Germany

**Keywords:** amphibian, captive nutrition, carotenoids, conservation breeding program, cutaneous microbiome, host–microbe interactions, rewilding, threatened species

## Abstract

The composition and dynamics of the skin bacterial and fungal microbiome is thought to influence host‐pathogen defence. This microbial community is shaped by host captivity, diet, and microbial interactions between bacterial and fungal components. However, there remains little understanding of how specific micronutrients influence bacterial and fungal microbiome composition and their inter‐domain interactions during rewilding of captive‐bred animals. This study experimentally investigated the effect of dietary beta‐carotene supplementation and subsequent field release on bacterial and fungal microbiome composition and dynamics using the Southern Corroboree frog (*Pseudophryne corroboree*) as a model system. We found large‐scale diversification of bacterial communities post‐release and similar diversification of fungal communities. The rewilded fungal mycobiome was more transient and demonstrated stronger temporal and micro‐spatial fluctuations than the bacterial microbiome. Accounting for temporal and spatial factors, we found strong residual associations between bacterial members, yet limited evidence for inter‐domain associations, suggesting that co‐occurrence patterns between bacterial and fungal communities are largely a result of shared responses to the environment rather than direct interactions. Lastly, we found supplementation of dietary beta‐carotene in captivity had no impact on post‐release microbiome diversity, yet was associated with approximately 15% of common bacterial and fungal genera. Our research demonstrates that environmental factors play a dominant role over dietary beta‐carotene supplementation in shaping microbiome diversity post‐release, and suggest inter‐domain interactions may also only exert a minor influence. Further research on the function and ecology of skin bacterial and fungal microbiomes will be crucial for developing strategies to support survival of endangered amphibian species.

## 1 Introduction

The skin acts as a vital barrier against pathogens and hosts diverse bacteria, fungi, eukaryotes, and viruses crucial for skin homeostasis and defence (Flowers and Grice 2020). Despite its importance, the skin microbiome is less explored compared to the gut microbiome, with limited knowledge about its ecological drivers, especially in relation to the fungal component (Ross, Rodrigues Hoffmann, and Neufeld 2019). Understanding the skin microbiome's ecology and function is particularly important within the context of wildlife conservation given the impact of cutaneous infectious diseases, such as the amphibian chytrid fungus, snake fungal disease, bat white‐nose syndrome, sarcoptic mange in mammals, and papillomavirus in birds and turtles (Escobar et al. 2022; Fisher and Garner 2020; Frias‐De‐Diego, Jara, and Escobar 2019; Leopardi, Blake, and Puechmaille 2015; Lorch et al. 2016). Resistance to many of these pathogens is linked to the skin microbiome (Bates et al. 2022; Swe et al. 2014; Vanderwolf et al. 2021), potentially through competitive exclusion and other interactions (McLaren and Callahan 2020) that are challenging to detect through co‐occurrence network analysis (Matchado et al. 2021). Some cutaneous infectious diseases have caused such extreme population declines that captive breeding programs are necessary conservation measures, leading to a focus on manipulating microbiomes in captive‐bred populations to promote health and species recovery (Dallas and Warne 2023).

The amphibian skin is used for respiration and osmoregulation and is particularly vulnerable to disease due to its permeable nature. Amphibian skin is covered with a sugar‐rich mucosal substrate that harbours a rich diversity of bacterial and fungal microbes, which together are hypothesised to be of functional importance to amphibian immunity and pathogen defence (Bates et al. 2022; Knutie et al. 2017; Kueneman et al. 2016; Walke and Belden 2016). Several studies have documented that captivity leads to a loss of skin microbial diversity across amphibian species (Bates et al. 2019; Kueneman et al. 2016, 2022). This loss of diversity may result in a decline in microbe‐mediated immunity (e.g., anti‐fungal properties; Kueneman et al. 2022), making captive amphibians more susceptible to pathogen infection (Bates et al. 2022; Kearns et al. 2017). There is also evidence that changes to skin microbiome composition can alter survival outcomes, for example, after pathogen exposure (Kueneman et al. 2016), although the downstream consequences for host immunity and fitness remain untested. As such, conditions during captivity and post‐release microbial dynamics may have microbiome‐mediated consequences for the success of ex situ conservation programs that rely on captive breeding and reintroductions.

While there appears to be a relatively consistent loss of bacterial diversity associated with captivity (Bates et al. 2019; Kueneman et al. 2022), it remains unclear the extent to which host experiences during captivity influence microbial composition, and whether such signatures persist after release. Moreover, fungal communities are an essential component of the amphibian skin microbiome (Kearns et al. 2017), yet very few studies have examined fungal community responses to captivity and rewilding. One exception is a study that examined the impact of captivity on the bacterial and fungal microbiome of two species of salamanders, which found captivity reduced both bacterial and fungal diversity similarly across both species, especially reducing the relative abundances of bacterial *Pseudomonas* and fungal *Cladosporium* (Bates et al. 2019). However, many amphibian conservation breeding programs raise offspring in captivity prior to release, where exposure to microbes across development is limited, and there remain few examples of how rewilding captive‐bred individuals affects skin microbial composition and dynamics.

As well as captivity per se, host factors such as differences in diet may also be important for mediating shifts in skin microbiomes during release. One group of essential nutrients that may be important for shaping the microbiome and host immunity are dietary carotenoids, a group of nutrients that are converted into Vitamin A and/or act as antioxidants, which together are crucial for immunity and colouration (Szuroczki, Koprivnikar, and Baker 2016, 2019; Weaver et al. 2018). Dietary carotenoid supplementation has previously been found to influence the amphibian skin bacterial microbiome (Edwards et al. 2017; Risely et al. 2023), and these effects might carry over once animals are released into the wild, as has been found with gut microbial communities in white‐footed mice (van Leeuwen et al. 2020). Yet, there remains very little understanding of the effects of specific carotenoids such as ꞵ‐carotene, and diet more broadly, on host‐associated microbiomes, both in amphibians and other vertebrates. Experimental and longitudinal studies that test how bacterial and fungal skin microbes respond to host diet manipulation, and examine how long these effects last, are crucial for improving our understanding of host‐microbiome interactions and developing effective ex situ conservation strategies.

Skin microbial dynamics and function may not only be shaped by host biology and environment, but might also be strongly influenced by microbial interactions (e.g., competition or mutualism; Gould et al. 2018; Spragge et al. 2023). Although interactions between bacterial members are known to be important for microbiome function (Spragge et al. 2023), we know much less about inter‐domain interactions, for example, between bacteria and fungi. Fungal microbiomes have been found to co‐vary with bacterial communities in cross‐sectional studies (Harrison et al. 2021; McKnight et al. 2022; Medina et al. 2019), raising the question as to whether observed associations between the bacterial and fungal microbiome are shared responses to host environment or the result of direct inter‐domain interactions, such as resource competition or cross‐feeding. Distinguishing between shared responses and direct interactions in microbiome studies is challenging, yet modelling methods that can identify residual correlations that remain after accounting for associations explained by known host and environmental factors can shed light on this relationship. This is because direct interactions between bacterial and fungal communities might reasonably be expected to generate detectable associations that persist independently from changes to host biology or environment.

In this study we examine the pre‐ and post‐release dynamics of the bacterial and fungal microbiome of captive‐bred Southern Corroboree frogs (*Pseudophryne corroboree*), a critically endangered montane frog from southeastern Australia that is primarily under threat from the amphibian chytrid fungus. Frogs were raised in captivity and subject to diets supplemented with one of four different dietary ꞵ‐carotene concentrations for 40 weeks following metamorphosis. ꞵ‐carotene was chosen because, alongside Lutein, it is the most abundant carotenoid found in the skin of captive *P*. *corroboree*. At 12 months post‐metamorphosis, metamorphs were released into outdoor mesocosms in their natural montane habitat. Skin bacterial and fungal communities were sampled just prior to release, and for a subset of frogs, samples were taken again at 2 months post‐release, and 1 year post‐release, in a mixed study design that includes both cross‐sectional and longitudinal sampling.

We aimed to quantify (1) long‐term shifts in diversity and taxonomic composition in bacterial and fungal microbiomes; (2) identify potential inter‐domain interactions between bacteria and fungi; and (3) identify whether dietary ꞵ‐carotene concentration impacts the bacterial and fungal microbiome and examine any carry‐over effects on the microbiome after field release.

## 2 Methods

### 2.1 Ethics

All procedures were approved by the University of Wollongong's Animal Ethics Committee (AE18/15), and New South Wales National Parks and Wildlife Service scientific licence number #SL102197.

### 2.2 Study Species

**FIGURE 1:**
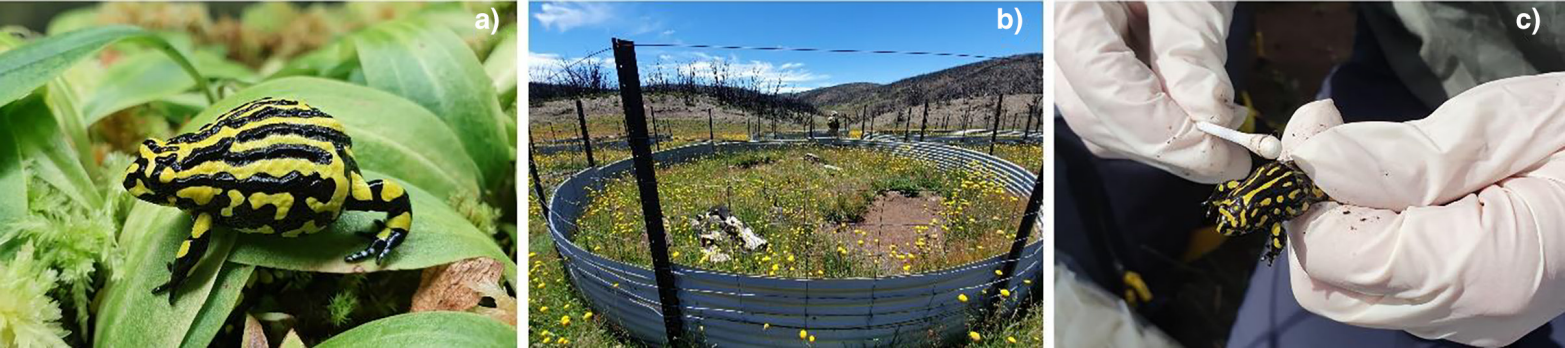
The study species and open‐air enclosures; (a) Adult Southern Corroboree frog, Pseudophryne corroboree, (b) Field‐release enclosures located in Kosciusko National Park, NSW, and (c) skin swab collection following rewilding and subsequent recapture in situ. Photographs courtesy of A.J. Silla.

The Southern Corroboree frog, *P*. *corroboree* (Figure 1a), is a terrestrial frog exclusively found in the sub‐alpine regions of Kosciuszko National Park, New South Wales. Nationally listed as Critically Endangered, the amphibian chytrid fungus has pushed the species to the brink of extinction (McFadden et al. 2018). A successful conservation breeding program, initiated in the late 1990s and involving multiple institutions, has resulted in a growing captive population. This population annually contributes offspring for reintroduction into the wild and supports ongoing conservation research (McFadden et al. 2018). In their natural habitat, juvenile and adult *P*. *corroboree* typically consume ants and other small invertebrates after metamorphosis, while tadpoles feed on algae and organic matter, and metamorphs under brumation over winter. Given that these prey items are rich in carotenoids, it is assumed that carotenoids naturally form a vital part of *P*. *corroboree'*s diet (Osborne 1991).

### 2.3 Study Animals

On February 5th, 2019, 136 *P*. *corroboree* metamorphs from eight clutches (A–G) were transported to the Environmental Research Centre (ERC) at the University of Wollongong (UOW) from a captive colony maintained at Taronga Zoo Sydney, where they were housed communally in groups of up to 25 individuals per terrarium. At the time of collection, individuals ranged in age from 4 to 8 weeks post‐metamorphosis.

### 2.4 Diet Treatments

Upon arrival at UOW, metamorphs were isolated, placed in individual enclosures measuring 21 cm × 12 cm × 12 cm, and randomly assigned to one of four experimental diet treatments. Each enclosure featured a 2 cm layer of aquarium‐grade pebbles, one cup of loosely packed sphagnum moss (*Sphagnum cristatum*), and a single small PVC pipe (inner diameter = 4.4 cm; length = 5.5 cm) for shelter. The sphagnum moss, obtained as a dehydrated brick, was rehydrated and rinsed several times with tap water prior to being thoroughly rinsed with Reverse Osmosis (RO) water before use. Enclosures underwent a comprehensive flush with RO water twice a week. To eliminate carotenoid residue and excrement, and prevent the buildup of ammonia, the sphagnum moss was replaced with fresh moss every 2 weeks.

Throughout the experiment, enclosures were housed in an artificially illuminated room with a controlled temperature of 20°C. Two UV‐B light bulbs (Reptisun 10.0 T5 High Output 36″ bulb; Pet Pacific, Australia) were suspended approximately 20 cm above the enclosures to supply ultraviolet‐B lighting. An automatic timer was employed to regulate the UV‐B lights, delivering 9 h of exposure each day. Furthermore, the frogs received natural ambient light from an external window, ensuring a seasonal photoperiod.

Before the initiation of experimental diets on March 15, 2019, all metamorphs were provided with a foundational diet comprising commercially available crickets (*Acheta domesticus*) for a period of 5 weeks. The experimental diets were formulated by dusting a standardised weight of 7–10‐day‐old crickets (15 g per treatment) with one of four treatment powders, corresponding to each unique experimental diet: 0 mg g^−1^ β‐carotene (C0); 1 mg g^−1^ β‐carotene (C1); 2 mg g^−1^ β‐carotene (C2); 3 mg g^−1^ β‐carotene (C3). Cellulose microcrystalline powder (435,236; Sigma‐Aldrich, Castle Hill, NSW) was incorporated into each dietary supplement at up to 3 mg g^−1^ to ensure a consistent mass of supplement and a uniform feed quantity across all treatments. Both basal and experimental diets were enriched with 0.25 g of calcium powder per feed, a measure implemented to prevent the occurrence of disorders associated with calcium deficiencies commonly observed in captive amphibians. Detailed information about the dietary supplements for each experimental treatment is available in Table S1. Throughout the study, individual frogs were fed 10–15 dusted crickets twice per week to be consumed ad libitum.

Individuals were maintained in captivity on experimental diet treatments for 40 weeks. On the 17th of December 2019, *P*. *corroboree* (*n* = 115) were transported from UOW to the reintroduction site.

### 2.5 Field Release

*Pseudophryne corroboree* were introduced into four specially designed circular open‐air enclosures (depicted in Figure 1b) constructed to release the species into a disease‐free environment. These enclosures were situated within the historical habitat range of *P*. *corroboree* in the sub‐alpine region of Kosciuszko National Park, NSW Australia. For conservation security reasons, specific location details of the field site are intentionally withheld. To maintain a ‘disease‐free’ status, precautions were taken to exclude other frogs, particularly the common eastern froglet *Crinia signifera*, known to serve as a reservoir host for the amphibian chytrid fungus (Brannelly et al. 2018). The ring enclosures were designed to mimic natural conditions as much as possible, providing the frogs with natural vegetation, refugia and microhabitats that the frogs would have historically been exposed to. The enclosures are open‐air, baseless, and placed over natural soils and vegetation, primarily the tussock grass *Poa sieberiana*. The ring enclosures contain naturally occurring prey items (small invertebrates such as ants, flies, beetles) and allow the movement of birds and other invertebrates. As such, while the movement of the frogs is restricted compared to wild frogs, the frogs are as far as possible still exposed to natural habitats and, with the exception of the common eastern froglet *Crinia signifera*, natural interactions with other species within the ecological community. Each circular open‐air enclosure was 6 m in diameter, with a 90 cm high galvanised corrugated iron fence dug 30 cm into the ground (as illustrated in Figure 1b). Within each enclosure, 10 wooden planks of varied sizes were randomly positioned to create refuge areas for the frogs and foster suitable structures for ant colonies—the primary diet of *P*. *corroboree*. Due to the abundant presence of ants and other small invertebrates in all enclosures, no supplementary feeding was necessary.

Prior to arrival at the field site, individuals from each diet treatment (C0, C1, C2 and C3) were assigned to a field enclosure (E1–E4) based on their pre‐release body weight (weight as of the 5th December 2019). This ensured that each diet treatment was equally represented within each field enclosure, and that there was no significant difference between enclosures in either the mean body weight of frogs (ANOVA: *F*_3,111_ = 0.1365, *p* = 0.9381) or the variance in body weight (Levene's test: *F*_3,111_ = 0.1724, *p* = 0.9149). Total number of frogs assigned to each of the field enclosures were as follows: enclosure 1 (E1): *n* = 29, enclosure 2 (E2): *n* = 29, enclosure 3 (E3): *n* = 28, enclosure 4 (E4): *n* = 29. At the time of reintroduction, frogs were approximately 12 months old post‐metamorphosis and weighed between 1.625 and 3.309 g (mean ± SEM = 2.415 ± 0.03 g).

### 2.6 Sampling of Cutaneous Microbiota

The cutaneous microbiota of each frog was sampled in captivity following 40 weeks on the experimental diets, just prior to field release. Approximately 2 months after field release (69 days), frogs were recaptured, sampled and returned to their field enclosures (Recap 1). Frogs were recaptured and sampled again 12 months after release (363 days; Recap 2). A major bushfire burned through Kosciuszko National Park in January 2020 (prior to T2), including the release site, and as a result only 37 of the 115 released frogs were recaptured during either T2 or T3.

During sample collection (Figure 1c), frogs were handled with latex gloves (Skinshield powderfree latex examination gloves, Livingstone) and gloves were changed between individuals. Immediately prior to sampling, frogs were rinsed once with 5‐mL sterile water (Baxter, Steripour) to remove transient microbiota. Cutaneous microbiota samples were collected using sterile rayon‐tip swabs (CLASSIQ swabs 167KS01, Copan Diagnostics, Inc., USA). Frogs were sampled by running a single swab tip across each of the following surfaces five times: dorsal (anterior to posterior), ventral (anterior to posterior), lateral (left and right sides), front legs from armpit to finger tips (left and right sides) and back legs from groin to the tip of the toes (left and right sides); 40 strokes per frog. Ten controls swabs were also taken to account for any contamination. Swabs were labelled and temporarily stored in a portable refrigerator (5°C) for approximately 24‐h before being stored in a −80°C freezer until they were analysed. In total, 178 swab samples, including 10 controls, were submitted for genomic analysis.

### 2.7 DNA Extraction, Amplification, and Sequencing

DNA extraction, amplification and sequencing were conducted by the Australian Genome Research Facility (AGRF). Briefly, DNA was extracted using a DNeasy power soil kit following manufacturer's instructions. For the bacterial microbiome, the V3–V4 region of the bacterial 16S rRNA gene was amplified using 341F (CCTAYGGGRBGCASCAG) and 806R (GGACTACNNGGGTATCTAAT) primers. For the fungal mycobiome, the ITS1‐2 region of the nuclear ribosomal internal transcribed spacer (ITS) fungal gene was amplified using the primers ITS1F (CTTGGTCATTTAGAGGAAGTAA) and ITS2 (GCTGCGTTCTTCATCGATGC). Thermocycling was completed with an Applied Biosystem 384 Veriti and using AmpliTaq Gold 360 (Life Technologies, Australia) for the primary PCR. The first stage PCR was cleaned using magnetic beads, and samples were visualised on 2% Sybr Egel (Thermo‐Fisher). A secondary PCR to index the amplicons was performed with TaKaRa Taq DNA Polymerase (Clontech). The resulting amplicons were cleaned again using magnetic beads, quantified by fluorometry (Promega Quantifluor) and normalised. The equimolar pool was cleaned a final time using magnetic beads to concentrate the pool and then measured using a High‐Sensitivity D1000 Tape on an Agilent 2200 TapeStation. The pool was diluted to 5 nM and molarity was confirmed again using a Qubit High Sensitivity dsDNA assay (ThermoFisher). This was followed by sequencing on an Illumina MiSeq (San Diego, CA, USA) with a V3, 600 cycle kit (2 × 300 base pairs paired‐end). Both bacterial and fungal reads were sequenced on the same run.

### 2.8 Bioinformatics

For the bacterial microbiome, sequence reads were processed using QIIME2 (v 2020.2; Bolyen et al. 2019). Sequences were merged, quality filtered, and chimera filtered using the DADA2 pipeline to generate amplicon sequence variants (ASVs; Callahan et al. 2016). This excluded 21% of reads. For the bacterial microbiome, primers were trimmed and reads were truncated at 277 (forward) and 220 (reverse) base pairs. ASVs were assigned a taxonomy using SILVA version 132 (Pruesse et al. 2007). A microbial phylogenetic tree was built using FastTree (Price, Dehal, and Arkin 2009). The phylogenetic tree was rooted with an Archaea sequence that was subsequently removed. Note that while Proteobacteria is now referred to as Pseudomonodota, and Actinobacteria is now referred to as Actinomycetota, we still refer to Proteobacteria and Actinobacteria in this study in order to be consistent with database taxonomy and figures.

For the fungal mycobiome analysis, paired‐end sequencing reads were also pre‐processed using the QIIME2 microbiome analysis pipeline (v 19.1; Bolyen et al. 2019) and DADA2 (Callahan et al. 2016) to remove dataset noise arising from artefacts and generate amplicon sequence variants (ASVs). This removed 35% of reads. Primers were trimmed and reads were truncated at 230 (forward) and 220 (reverse) base pairs. For a taxonomic assignment, we used the UNITE database release 16.10.2022 (Abarenkov et al. 2010). Before building the classifier we de‐replicated the database using the dereplicate plugin of Rescript (Robeson et al. 2021) in QIIME2. After de‐replication, a classifier was built and taxonomy was assigned. We added an Opisthokonta sequence (*Fonticula alba*) to root the phylogenetic tree, which was constructed using FastTree (Price, Dehal, and Arkin 2009). The root was subsequently removed.

### 2.9 Data Processing

Sample filtering: After quality filtering and merging, the median read count was 15,117 and 12,115 reads for bacterial and fungal data, respectively. For the bacterial microbiome data, samples were excluded if they had a sequencing depth under 2000 reads. For the fungal mycobiome data, samples were excluded if they had a sequencing depth under 1000 reads. This difference in filtering threshold was justified using rarefaction curves (Figure S1), which showed that 2000 and 1000 reads largely captured sample diversity for bacterial and fungal communities, respectively. These steps removed 53 and 41 samples from the bacterial and fungal datasets, respectively. We also removed one sample taken from natural enclosure four, as there was only one sample representing this enclosure, reducing statistical power. The final sample size for analyses on the bacterial microbiome was 124 samples, and the final sample size for fungal analyses was 136 samples. Of these, 109 samples had paired bacterial and microbiome data.

#### 2.9.1 Amplicon Sequence Variant Filtering

Amplicon sequence variants were filtered if they were not bacteria or fungi, not assigned to a phylum (as these are assumed to be spurious), or if they were classified as mitochondria or chloroplasts. Potential contaminant ASVs were identified using 10 control samples and the *decontam* package (Davis et al. 2018), applying the prevalence methods and a threshold parameter of 0.2. Across the whole dataset, we detected 10,622 bacterial ASVs and 1687 fungal ASVs.

#### 2.9.2 Normalisation

For all alpha and beta diversity analyses presented, data were rarefied to 2000 reads (bacteria) and 1000 reads (fungi). This was justified by examining rarefaction curves (Figure S1). For genus‐level analyses, data was Centre Log Ratio (CLR) transformed. We elaborate on the specifics of data manipulation for specific analyses below.

### 2.10 Data Analysis

#### 2.10.1 The Effect of Field Release on the Bacterial and Fungal Microbiome

As well as visualising changes to genus‐level relative abundances using heatmaps and barplots, we modelled the effect of field release (pre‐release vs. post‐release communities) using a joint modelling approach using the function *gllvm::gllvm* (Niku et al. 2019), specifying two latent variables, a Gaussian distribution, and including frog ID as a random effect. GLLVMs are superior to methods such as differential abundance analysis because they model joint responses of species to explanatory variables and aim to tease apart the causes of species co‐occurrence. Prior to fitting the model, raw microbial counts were agglomerated to genus level, CLR transformed and filtered to include only the 25 most abundant genera per bacterial and fungal dataset (50 in total). Rarer taxa are challenging to model robustly, and these 50 genera accounted for 64% and 87% of all bacterial and fungal reads, respectively. At this point, bacterial and fungal datasets were merged so they could be modelled together. Quality checks on model fit were performed to ensure model assumptions were met.

To examine the effect of field release on alpha diversity, we fitted generalised linear mixed models on rarefied data to quantify the effect of time points on two measures of alpha diversity (within individual diversity): observed ASV richness and Shannon index. Linear models were fitted using *glmmTMB::glmmTMB* and included time point and sequencing depth as fixed effects and frog ID as a random effect, and modelled with a Gaussian distribution. Bacterial and fungal data were analysed separately yet treated identically.

To examine the effect of sample time point on beta diversity we analysed community composition based on rarefied data and using a weighted Unifrac distance matrix, which accounts for abundance and microbial phylogeny. We modelled associations between distances and sample variables using a distance‐based redundancy analysis (dbRDA; a type of constrained ordination) and applied using *vegan::capscale* (Jari Oksanen et al. 2018). The dbRDA model included time point and sequencing depth as co‐variates. The model was summarised using *vegan::anova*.*cca*. We tested for differences in interindividual variation between time points using a beta dispersion test (*vegan::betadisper*) on the weighted Unifrac distance matrix which measures average distances of points from the group centroid.

#### 2.10.2 Quantifying Microbial Interactions Through Association Networks

We built association networks from a GLLVM model (Niku et al. 2019), similar to that described above. We included both time point and enclosure ID in the model, to account for all sources of known environmental variation. We did not include frog ID as a random effect in this model due to problems with model convergence, but note that the results of this model were almost identical to a model simply including status (pre‐ vs. post‐release) and frog ID as a random effect, suggesting individual ID explained little to no variation in microbiome composition. We compared the model explanatory power to a null model without explanatory variables in order to decompose sources of variation in taxa associations. Residual correlations were extracted from the GLLMV model using the *gllvm::getResidualCor* function. We did not build separate networks for pre‐ and post‐release communities because we believe these would be misleading: sources of (unmeasured) variation will be much larger for frogs in outdoor enclosures than in the lab, and differences in network diversity can strongly bias measures of network structure (Fründ, McCann, and Williams 2016). Therefore, separate networks would be largely incomparable given the microbial networks for rewilded frogs are much larger and by default more complex than the low‐diversity network of lab frogs, and comparing them could lead to biased conclusions.

#### 2.10.3 Testing the Effect of ꞵ‐Carotene Treatment on Bacterial and Fungal Communities

We explicitly examined the effect of ꞵ‐carotene treatment prior to field release on alpha diversity, beta diversity, and the abundances of common genera using the methods described above. We analysed pre‐ and post‐release communities separately due to the large difference in their microbial communities. In addition, to test the effect on genera‐level abundances we increased the number of genera examined from the top 25 to the top 40, thereby including 80 genera (40 bacteria and 40 fungi) in our GLLVM models.

## 3 Results

### 3.1 Complete Taxonomic Restructuring of Bacterial and Fungal Microbiomes on Field Release

**FIGURE 2:**
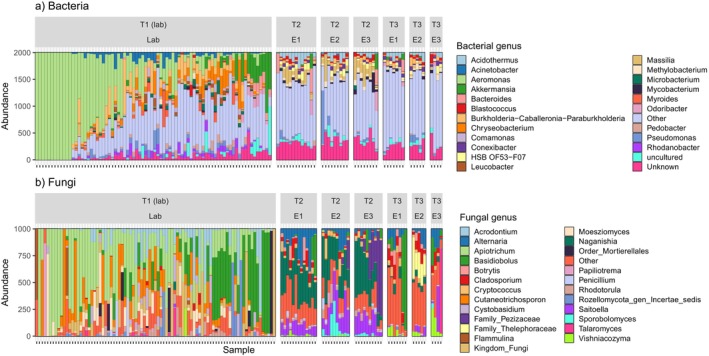
Genus‐level changes in the relative abundance of (a) bacterial and (b) fungal components of the skin microbiota of Southern Corroboree frogs before and after release. Samples are separated by time point (T1–T3) and lab/enclosure (lab; E1–E3).

Both the bacterial microbiome and fungal mycobiome underwent complete taxonomic restructuring after field release, with only 4% of bacterial and 5% of fungal ASVs shared between pre‐ and post‐release frogs. At the phylum level, pre‐release bacterial microbiomes were strongly dominated by Proteobacteria (61% relative abundance), while post‐release microbiomes were dominated by Actinobacteria (36%) and Proteobacteria (37%; Figure S2a). The fungal mycobiome of pre‐release frogs was dominated by the phyla Basidiomycota (56%) and Basidiobolomycota (16%; Figure S2b). After field release, Ascomycota replaced Basidiobolomycota and other rarer fungal taxa to become the dominant phylum (46%), alongside Basidiomycota (44%; Figure S2b). At the genus level, bacterial *Aeromonas* was replaced by rarer taxa after soft release (Figure 2a), while for fungal genera, *Apiotrichum* was replaced by either *Naganishia* or *Alternaria* (Figure 2b).

**FIGURE 3:**
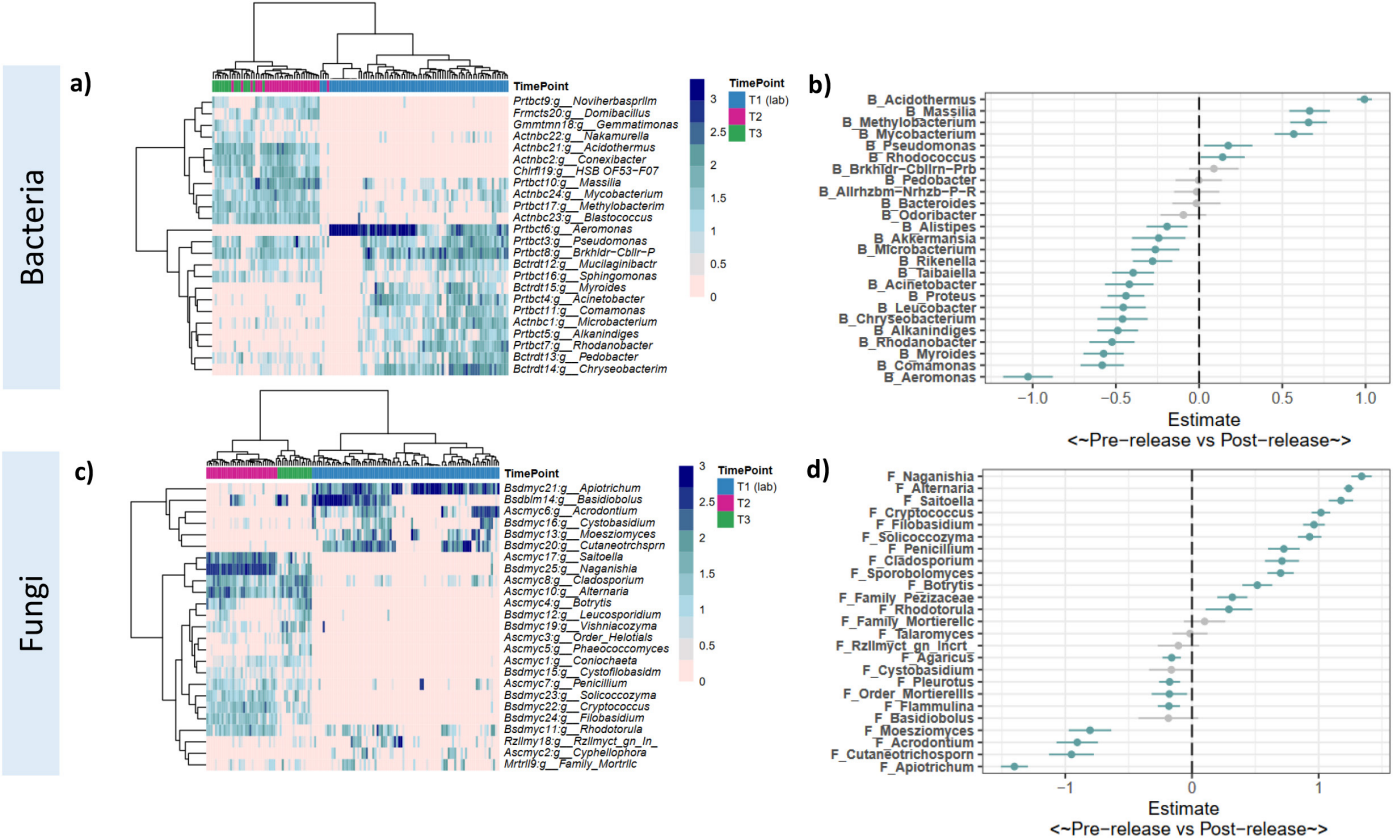
Genus level differences in the microbiota of the Southern Corroboree frog between pre‐ (T1) and post‐release (T2 and T3) for the 25 most abundant bacterial genera (top panels) and the 25 most abundant fungal genera (bottom panels). (a) Clustered heatmap showing sample‐level log‐transformed relative abundances of common bacterial genera and (b) fungal genera. Genera names are preceded by the phyla they belong to, and samples are coloured by the time point they were collected at; (c) Results from a general linear latent variable (GLLVM) model on CLR‐transformed counts identifying genera that differ between pre‐ and post‐release communities (positive estimates represent genera that are more common in post‐release frogs) for bacterial genera and (d) fungal genera. Error bars represent 95% confidence intervals, and significant associations are coloured blue.

When considering the most abundant 25 bacterial and fungal genera, clustered heatmaps indicate strong clustering within a time point, but stark changes in composition between time points (Figure 3a,b). These patterns in relative abundance were reflected by general linear latent variable models (GLLVM), indicating that field release led to the replacement of bacterial genera *Aeromonas* and *Comamonas* by *Acidothermus* and *Massilia* (Figures 2a and 3c), and by the replacement of fungal genera *Apiotrichum* and *Cutaneotrichosporn* by *Naganishia* and *Alternaria* (Figures 2b and 3d).

### 3.2 Field Release Increases Bacterial and Fungal ASV Richness

**FIGURE 4:**
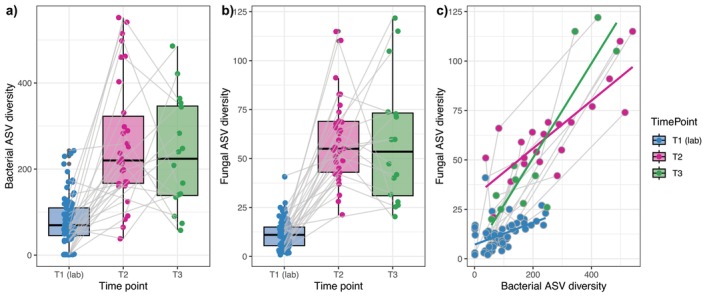
ASV richness of (a) bacterial and (b) fungal communities of Southern Corroboree frog skin before (T1) and after (T2 and T3) field release; (c) the correlation between fungal and bacterial ASV diversity. Lines connect longitudinally sampled individuals.

After field release, bacterial ASV diversity increased by 300% (from an average of 80 to 245 ASVs; linear model: *z* = 10.7, *p* < 0.001, adj. *R* ^2^ = 0.49; Figure 4a) and fungal ASV diversity increased by 500% (from an average of 11 to 60 ASVs; linear model: *z* = 16.8, *p* < 0.001, adj. *R* ^2^ = 0.7; Figure 4b). These increases occurred at the individual level, given frogs were sampled longitudinally (Figure 4a,b). In contrast, there was no change in bacterial or fungal ASV diversity between the two sampling points post‐release, suggesting that at 2 months post‐release, skin microbial diversity had peaked and remained thereafter. Results were similar when examining the impact of time point on Sharon diversity (bacteria: linear model: *z* = −10.2, *p* < 0.001, *R* ^2^ = 0.54, Figure S3a; fungi: linear model: *z* = −10.2, *p* < 0.001, *R* ^2^ = 0.55, Figure S3b). Individual bacterial and fungal ASV diversity was highly positively correlated across all time points (linear model: *z* = 16.5, *p* < 0.001, *R* ^2^ = 0.8; Figure 4c).

### 3.3 Post‐Release Fungal Communities Are More Dynamic Than Bacterial Communities

**FIGURE 5:**
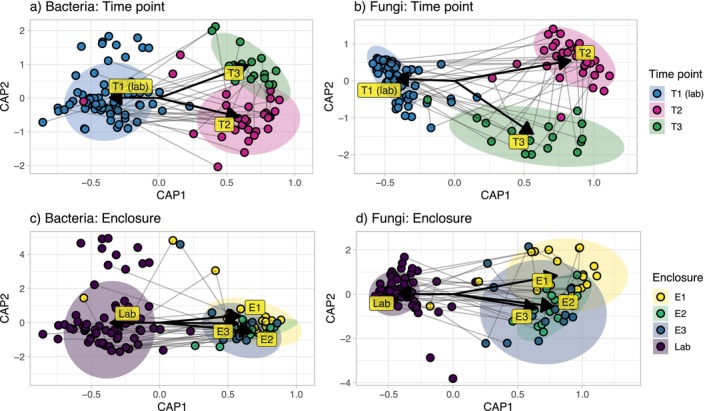
Constrained ordinations of cutaneous microbial community composition of the Southern Corroboree frog before (T1) and after (T2 and T3) field release, demonstrating clustering of community composition by time point (a, b) and enclosure (c, d), for bacterial (a, c) and fungal (b, d) communities. Lines connect longitudinally sampled individuals. Distances are calculated based on weighted Unifrac.

The effects of time point on bacterial and fungal whole‐community dynamics were assessed through constrained beta diversity analyses (Figure 5). Both bacterial (Figure 5a; ANOVA on a dbRDA: *F* = 19.0, *p* < 0.001) and fungal (Figure 5b; ANOVA on a dbRDA: *F* = 28.6, *p* < 0.001) communities underwent near‐complete changes in whole‐community composition after field‐release. There was minimal impact of enclosure ID on bacterial and fungal microbiomes, as bacterial communities did not cluster by enclosure post‐release (Figure 5c), although fungal communities did demonstrate weak clustering (Figure 5d). While the skin microbiomes of pre‐release frogs had lower alpha diversity, they did show higher inter‐individual variation than the microbiomes of post‐release frogs, as measured by a beta dispersion test (bacteria: ANOVA on a dispersion matrix, *F* = 11.0, *p* < 0.001; fungi: *F* = 9.7, *p* < 0.001).

The fluctuations in fungal communities across time points and enclosures were the result of localised invasions of particular genera. For instance, the fungal genera *Naganishia* and *Saitoella* were common 2 months post‐release (T2), yet largely absent at 1 year post‐release (T3), while the genus V*ishniacozyma* demonstrated the opposite pattern. A genus from the fungal family *Thelephoraceae* was also common in one particular enclosure 1 year post‐release, as was a genus from the family *Pezizaceae*, yet largely absent from other enclosures and at other time points (Figure 2).

### 3.4 Limited Residual Inter‐Domain Associations

**FIGURE 6:**
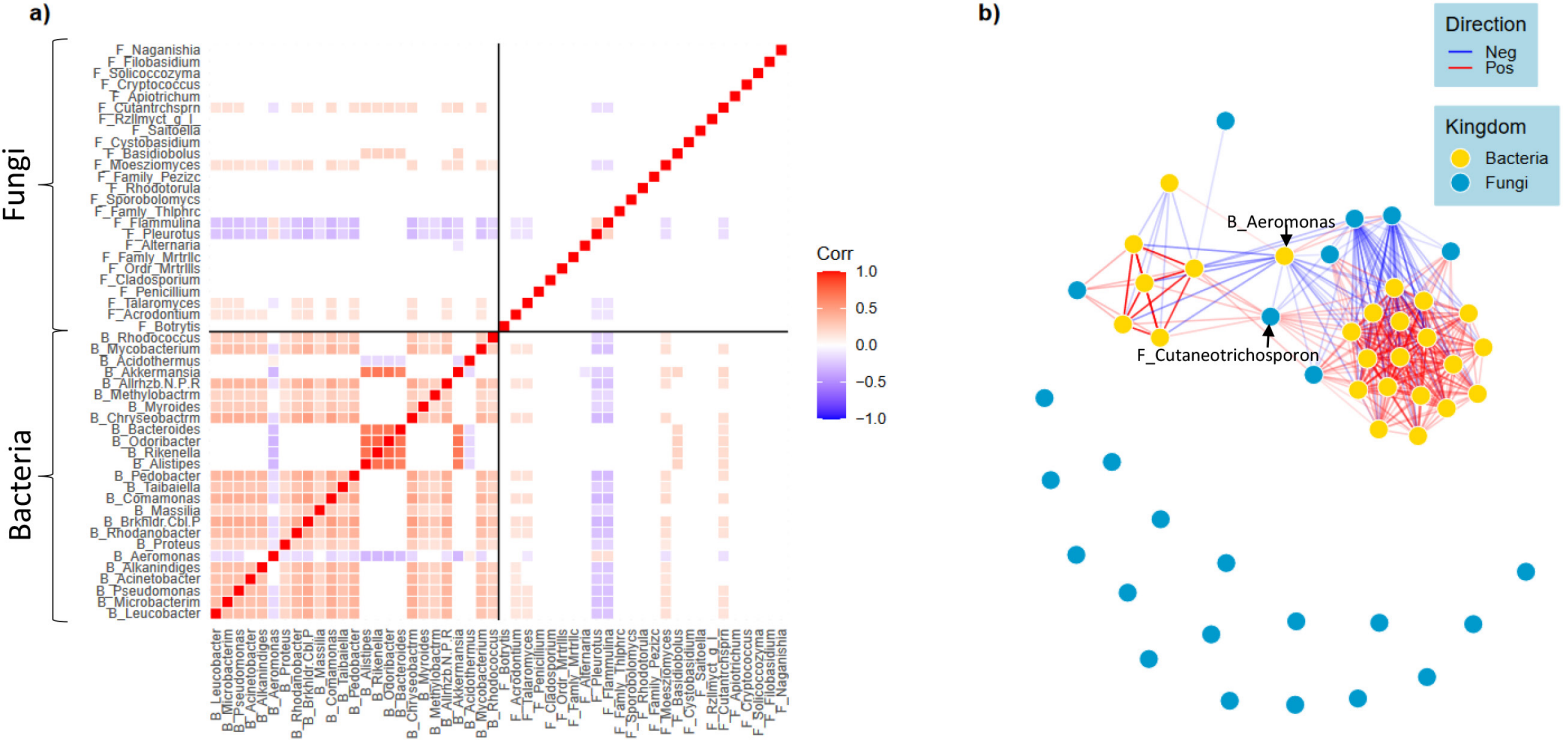
Associations between the 25 most abundant bacterial and fungal genera within the cutaneous flora of Southern Corroboree frogs; (a) A residual correlation matrix generated from a GLLMV model predicting associations between the 25 most abundant bacterial (“B” prefix) and fungal (“F” prefix) genera, accounting for release status and time point; (b) The same residual association network based on the residual correlation matrix shown in (a), but visualised in network format. Bacteria are coloured yellow and fungi are coloured blue. Negative associations are represented by blue, and positive association are represented by red. See Figure S5 for node names.

Across the 25 most common bacterial and fungal genera, co‐occurrence networks were developed by applying general linear latent variable models on CLR transformed data (Figure 6). Our model indicated that strong correlations between almost all taxa existed when not accounting for temporal and spatial factors (Figure S4). Of these associations within the network, 68% were explained by differences in time and enclosure, and 32% were independent or due to unmeasured variation in environmental or host factors. When accounting for shared responses to temporal and spatial factors, we found strong residual and positive associations between most bacterial genera, but very few residual associations between fungal genera, and limited inter‐domain associations (Figure 6a,b). We found the strongest independent inter‐domain associations between two fungal genera, *Flammulina* and *Pleurotus*, which had strong negative associations with a cluster of wild‐associated bacteria, as well as the fungal genus *Cutaneotrichosporn*, which was positively associated with bacterial clusters (Figure S5).

### 3.5 β‐Carotene Treatment Affects Specific Microbial Genera But Not Diversity

Dosage of β‐carotene prior to field release had no significant effect on alpha diversity (Table S2a) or beta diversity (bacterial: *F* = 1.05, *p* = 0.38; fungal: *F* = 0.85, *p* = 0.66) in frogs pre‐release. There was also no effect on alpha diversity (Table S2b) or beta diversity (bacterial: *F* = 1.3, *p* = 0.15; fungal: *F* = 0.71, *p* = 0.86) post‐release.

We next examined the influence of β‐carotene treatment on the abundances of specific genera, limiting our analyses to the 80 most common bacterial and fungal genera (40 bacterial and 40 fungal genera) pre‐ and post‐release. Due to the restructuring of the microbial community following field release, the most common 80 genera were largely non‐overlapping between the two groups, yet 19 genera were shared, including *Pseudomonas*, *Massilia*, *Mycobacterium*, *Akkermansia* (bacteria), and *Penicillium*, *Cladosporium*, *Saitoella*, and *Nagishia* (fungi).

**FIGURE 7:**
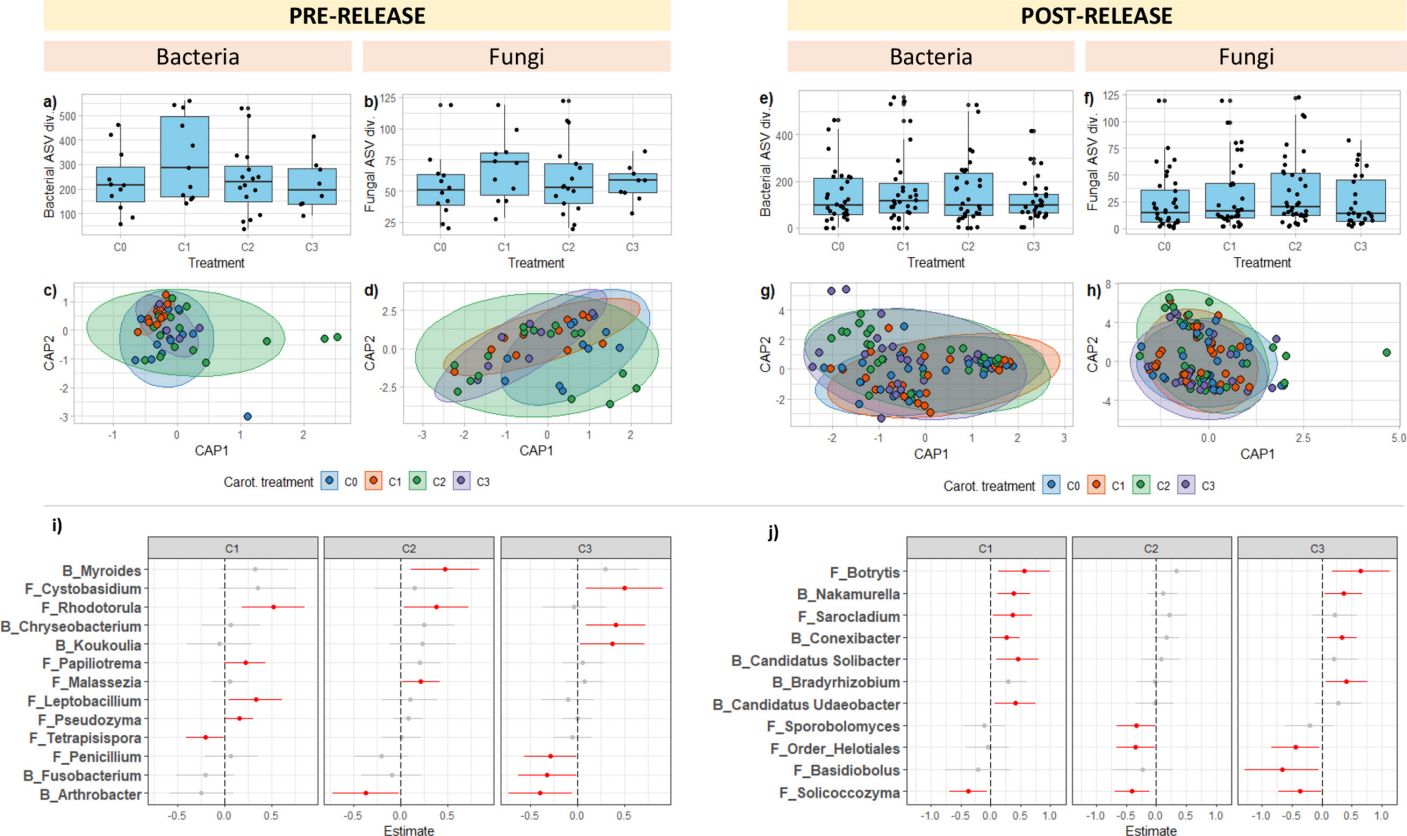
The effect of ꞵ‐carotenoid treatment on alpha diversity (top panels), beta diversity (middle panels), and genus‐level abundances (bottom panels) of the skin microbiota of Southern Corroboree frogs, split by samples taken pre‐ and post‐release; (a) Observed ASV richness across treatment groups for bacterial taxa and (b) fungal taxa in pre‐release frogs; and (c) beta diversity of bacterial communities and (d) fungal communities based on a constrained ordination of weighted Unifrac distances coloured by carotenoid treatment. (e–h) The same as (a) to (d) but for samples taken from post‐release frogs; (i, j) Estimates and 95% confidence intervals of genus‐level associations with carotenoid treatment for samples taken (i) pre‐release and (j) post‐release. Significant associations are highlighted in red.

Β‐carotene treatment influenced the abundance of 16% of common genera pre‐release (Figure 7i), and 13% of genera post‐release (Figure 7j). None of the genera that were significantly associated with β‐carotene treatment were shared across pre‐ and post‐release samples. Pre‐release, β‐carotene supplementation was associated with increased abundance of *Myroides* (bacteria) and *Cystobasidium* (fungi), and decreased abundances of *Arthrobacter* (bacteria) and *Penicillium* (fungi). Post‐release, β‐carotene supplementation was associated with increased the abundance of *Nakamurella* (bacteria) and *Botrytis* (fungi), and decreased abundances of *Solicoccozyma* and *Basidiobolus* (both fungi). Β‐carotene dosage did not appear to impact effect sizes, with both effect direction and size being largely consistent across dosage and genera.

## 4 Discussion

This study examined the combined effects of field release and β‐carotene treatment on the skin bacterial and fungal communities of captive bred Southern Corroboree frogs. We found that, after field release, both bacterial and fungal communities diversified by 300% and 500%, respectively, and there was a strong positive correlation between bacterial and fungal richness at the sample level. Fungal communities were more sensitive to temporal and micro‐spatial structuring than bacterial communities, with distinct differences in fungal community dynamics between release enclosures, despite their very close proximity to each other. Although diversity increased dramatically post‐release, the skin microbiome became more homogenous across individuals, with higher inter‐individual variation observed in lab frogs than those in outdoor enclosures. While bacterial taxa demonstrated strong residual associations with each other, most associations between bacteria and fungi were explained by temporal and spatial structuring, suggesting limited interactions between bacterial and fungal communities. Lastly, we found that β‐carotene treatment had no impact skin microbial diversity but did influence the abundances of specific genera, although the identity of affected genera differed between pre‐ and post‐release communities.

Our findings of increased skin microbial diversity following host rewilding are consistent with studies comparing wild and captive amphibians (Bates et al. 2019; although see Becker et al. 2014; Fieschi‐Méric et al. 2023; Kueneman et al. 2022). However, it remains unclear whether taxonomic shifts with captivity and rewilding can be generalised across host phylogenies or whether responses are largely species‐specific. In this study we found that taxa belonging to *Aeromonas* and *Apiotrichum* dominate the bacterial and fungal microbiome of captive Southern Corroboree frogs, respectively. *Aeromonas* is a genus that contains many opportunistic skin pathogens of amphibians, such *as A*. *hydrophila*, *A*. *veronii*, *A*. *sobria*, and *A*. *salmonicida* (Khalifa and Bekhet 2018; Miller et al. 2008; Pastorino et al. 2023), while *Apiotrichum*, previously known as *Trichosporon*, also contains opportunistic pathogens (Lo et al. 2021). Following field release, both *Aeromonas* and *Apiotrichum* were almost completely eliminated from the microbial community, replaced instead by *Acidothermus*, *Massilia*, and *Methylobacterium* (bacteria), and *Naganishia*, *Alternaria*, and *Saitoella* (fungi). Whether these taxa respond similarly to captivity and rewilding in other species is unclear due to differences in methods, reporting, and study designs across studies. Nevertheless, taxa such as *Pseudomonas* (bacteria) and *Cladosporum* (fungi) increased in relative abundance following field release which is consistent with a study that examined the impact of captivity on salamanders (Bates et al. 2019). Most *Pseudomonas* phylotypes were also more common in wild rather than captive newts (Fieschi‐Méric et al. 2023), suggesting there may be some consistent taxonomic shifts upon captivity and rewilding across host species. Meta‐analyses that examine taxonomic and broad functional responses to captivity and rewilding across host species will be required to identify whether taxonomic shifts are generalizable or host species‐specific.

We found that bacterial communities were less sensitive to temporal and micro‐spatial fluctuations in taxonomic composition than fungal communities, and that the bacterial microbiome did not change considerably between 2‐months and 1 year post‐release. A previous longitudinal study on how field release influences the bacterial microbiome of boreal toads, conducted over 2 months, showed that the taxonomic composition of the microbiome stabilised at approximately 1 month post‐release (Korpita et al. 2023), and longitudinal dynamics were similar across the individual toads sampled. As the first post‐release sampling in this study was conducted at 2 months, it is reasonable to assume that the bacterial microbiome had stabilised by this point. However, it is important to note that a natural disturbance event occurred just prior to the first sampling event post‐release in the form of a wildfire. The fact that bacterial communities did not change much between 2 months and 1 year post‐release, despite regeneration of natural vegetation over this time, suggests the taxonomic composition of this community is remarkably stable even in the face of habitat change.

While the taxonomic composition of the bacterial microbiome of post‐released frogs in this study was rather similar across individuals, other longitudinal studies of wild amphibians have shown this community to be relatively dynamic yet still maintaining inter‐individual differences (Ellison, Knapp, and Vredenburg 2021), and subject to predictable seasonal changes (Ellison, Knapp, and Vredenburg 2021; Walke et al. 2021). Much less is known about the fungal microbiome; however, and our study suggests that this community is far more dynamic and prone to temporary fungal invasions than the bacterial microbiome. This may explain the lack of associations between fungi and bacteria found in this study. The consequences of these differences in dynamics are unclear, but they draw attention to the possibility that amphibians maintain tighter immune control over their skin bacterial microbiome than the fungal mycobiome. If so, one would expect higher levels of phylosymbiosis between hosts and their bacterial microbiomes compared to their fungal mycobiomes, yet this remains to be tested.

Bacterial and fungal microbiomes often covary (Harrison et al. 2021; McKnight et al. 2022), raising the question as to whether inter‐domain interactions influence microbiome structure in amphibians independently of host and environmental factors. Such interactions may be important for mediating host fitness outcomes, as they are within bacterial microbiomes (Gould et al. 2018), yet we know little about bacteria‐fungi interactions outside of laboratory conditions (Deveau et al. 2018). While we found that bacterial and fungal taxa co‐varied, most of this co‐variance was explained by spatial and temporal changes experienced by the host (e.g., due to field release). There was limited evidence for residual correlations between bacterial and fungal taxa that might be expected if they influenced microbiome structure independently of host and environmental factors.

We found the strongest inter‐domain interactions involved two fungal genera, *Flammulina* and *Pleurotus*, which had strong negative associations with a cluster of wild‐associated bacteria, as well as the fungal genus *Cutaneotrichosporon*, which was positively associated with many bacterial clusters. Both *Flammulina* and *Cutaneotrichosporon* may have anti‐microbial properties more broadly, as some members of *Flammulina* have been found to inhibit the ability of pathogenic fungi to adhere to host cells (Kashina et al. 2016), while *Cutaneotrichosporon moniliiforme* has been linked to host resistance to White Nose Syndrome in bats (Vanderwolf et al. 2021). However, while these fungal genera may potentially demonstrate anti‐microbial properties, both were differentially more abundant in captive frogs than rewilded frogs in this study (Figure 3). Whether this reflects the possibility that captive frogs are more likely to harbour ‘core’ beneficial taxa that may be vertically inherited (e.g., through egg spawn), or, conversely, that diverse microbiomes upon rewilding may actually increase susceptibility through increased exposure and exclusion of beneficial taxa, remains unclear—although evidence suggests higher diversity of the skin microbiome is generally beneficial (Harrison et al. 2019; Vanderwolf et al. 2021).

We found no effect of β‐carotene treatment on microbial diversity although there were some associations with specific genera, which were largely independent of β‐carotene concentration. A previous study on captive individuals of this species found that supplementation with a broad‐spectrum dietary carotenoid had long‐lasting effects on the skin bacterial microbiome and diversity (Risely et al. 2023), and cultured bacteria (Edwards et al. 2017), suggesting that other types of carotenoids or their combined effects, rather than specifically β‐carotene, have a stronger effect on the amphibian skin microbiome. The broad‐spectrum carotenoid supplement used in these previous studies included additional carotenoids such as Astaxanthin, Alpha Carotene, Beta Cryptoxanthin, Zeaxanthin, Lutein, and Lycopene. Some of these carotenoids (including β‐carotene) are converted into vitamin A, which is involved in multiple physiological processes, including immunity, reproduction, development, and metabolism (Clugston and Blaner 2014), while other carotenoids act as antioxidants. Limitations in specific types of dietary carotenoids may affect the normal secretion of innate immune compounds, such as defensins and lysozymes, that may exert some control over the skin microbiota (Colombo et al. 2015; Woodhams et al. 2023). If such effects on host immunity are long‐lasting, then this could act as a mechanism by which β‐carotene treatment during captivity impacts newly introduced skin microbes after rewilding.

Our study provides rare evidence on how factors such as diet supplementation during captivity shape microbial composition and dynamics post‐release. However, the effect of β‐carotene supplementation on microbial composition was rather weak, even in captive animals, and only impacted taxa that were subsequently eliminated or became very rare in released hosts. Further studies that manipulate the microbiota more strongly during captivity (e.g., through broad=spectrum carotenoids, antibiotics, altered terrarium substrates, or whole‐diet manipulation) may be better placed to examine carry over effects on the microbiota and host health after animals are released. Microbial disturbance due to pathogen infection has been shown to persist for at least 48 days post‐infection, even after pathogen clearance (Jani et al. 2021), yet it is unknown whether such signatures would remain after rewilding. This may be expected given the almost complete restructuring of amphibian skin microbiome shown here and in other studies (Bates et al. 2019; Kueneman et al. 2022).

Our findings provide valuable insights into the processes that influence the structuring of bacterial and fungal skin microbial communities in amphibians during rewilding. Our results suggest that rewilded fungal microbiomes are less diverse yet more dynamic than bacterial microbiomes, with these domains demonstrating limited interactions with each other. Our finding that β‐carotene treatment only has weak and taxa‐specific effects on the skin microbiome indicate that, while these effects may still have downstream consequences for host fitness, future research on microbiome‐mediated fitness effects in amphibians should focus on other dietary compounds that are likely to vary in availability in natural settings. The consequences of our findings for host health and fitness remain unclear, as we still have a relatively poor understanding of skin bacterial and fungal function in amphibians. Although specific microbial taxa have been linked to host pathogen susceptibility, studies that link taxonomic composition to both functional composition and host physiological outcomes, including but not limited to pathogen resistance, are sorely needed to improve our understanding of the downstream consequences of skin microbiome variation.

## Author Contributions

A.J.S. and P.G.B. conceived the study, acquired funding, obtained animal ethics approval, and were responsible for frog husbandry. D.A.H. designed and managed construction of the release enclosures and obtained release permits. P.G.B. and D.A.H. released the frogs and D.A.H. recaptured them. A.J.S. collected the skin swabs pre‐ and post‐release and obtained the data. DNA extraction, amplification and sequencing was outsourced to the Australian Genome Research Facility (AGRF) with methods and taxonomic coverage guided by A.R. and B.J.H. in consultation with P.G.B. and A.J.S. A.R. and A.S.C. processed the data. A.R. and B.J.H. directed and performed the data analysis. A.R. wrote the manuscript with input from all authors.

## Conflicts of Interest

The authors declare no conflicts of interest.

### Open Research Badges

This article has earned Open Data, Open Materials and Preregistered Research Design badges. Data, materials and the preregistered design and analysis plan are available at [[insert provided URL(s) on the Open Research Disclosure Form]].

## Supporting information


Data S1.

## Data Availability

16S and ITS sequences are deposited on NCBI under SRA BioProject PRJNA1065914 (https://www.ncbi.nlm.nih.gov/bioproject/?term=PRJNA1065914). Processed data and code are available at https://zenodo.org/records/13173354 (Risely 2024).
