# Supplementary material for: Skin Bacterial and Fungal Microbiome Responses to Diet Supplementation and Rewilding in the Critically Endangered Southern Corroboree Frog
Source: Mol Ecol. 2024 Oct 21;33(23):e17562. doi: 10.1111/mec.17562 (PMC11589661; doi:10.1111/mec.17562)
Supplement: Supplementary file 1 — Data S1. [file MEC-33-e17562-s001.docx]

**SUPPLEMENTARY MATERIALS**

Skin bacterial and fungal microbiome responses to diet supplementation and rewilding in the critically endangered Southern Corroboree frog

Alice Risely, Phillip G. Byrne, David A, Hunter, Ana S. Carranco, Bethany J. Hoye, and Aimee J. Silla

Supplementary figures: S1 to S5

Supplementary tables: S1 to S2

**Supplementary figures**

Fig. S1) Rarefaction curves by sequencing depth for bacterial (left) and fungal (right) communities for the skin microbiome of the Southern Corroboree frog. Vertical lines represent levels of rarefaction for each dataset.


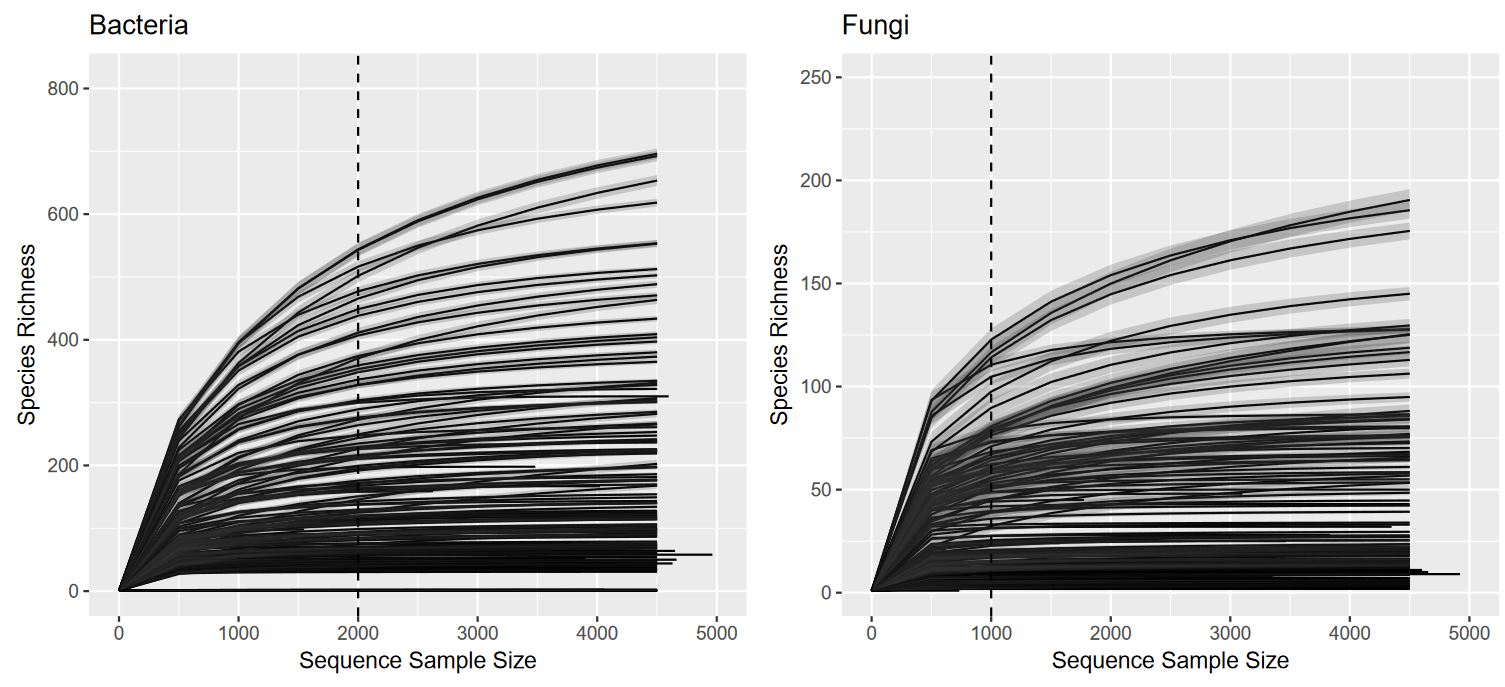


Fig. S2) Relative taxonomic composition of Southern Corroboree frog skin flora at the phylum level of a) bacteria and b) fungi across before release (T1), 2 months after release (T2) and 12 months after release (T3) across three soft-release enclosures (E1-3).**
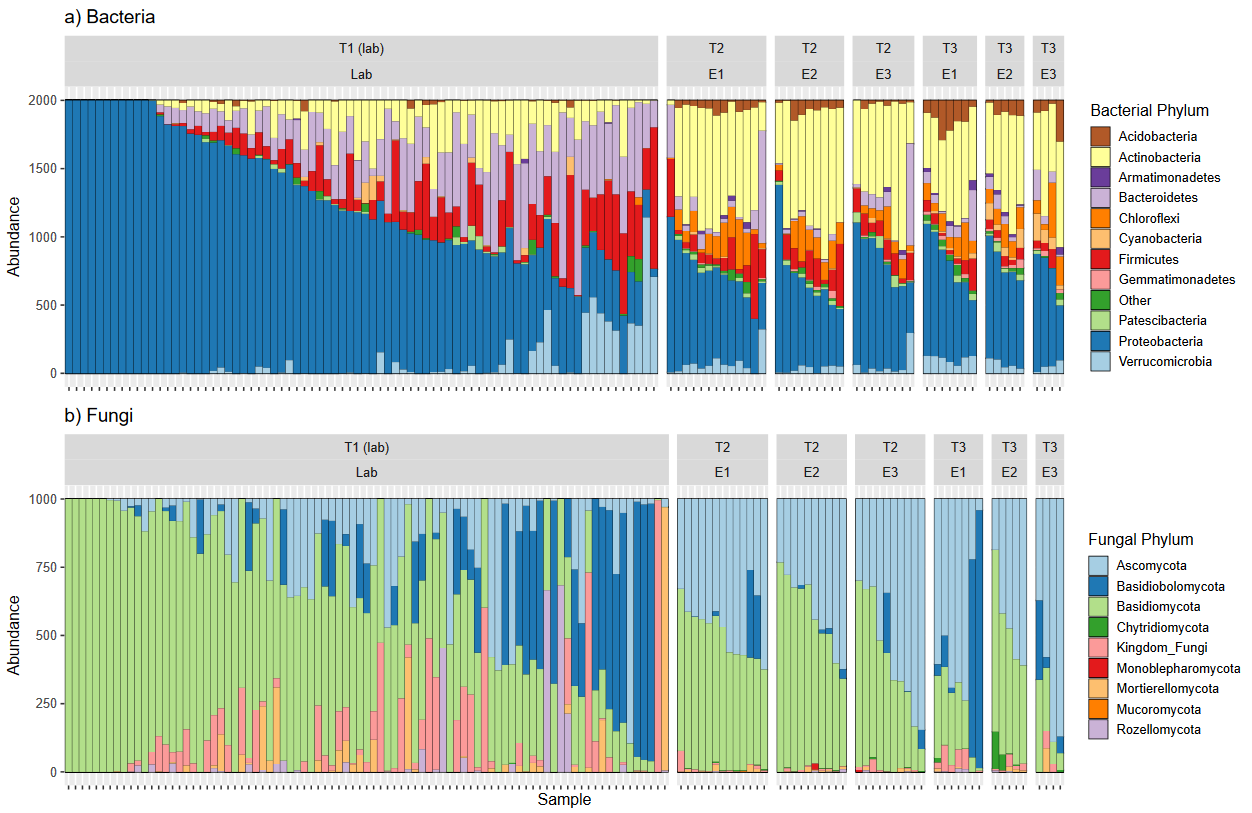
**

Fig. S3) Shannon diversity of a) bacterial and b) fungal skin microbiomes of the Southern Corroboree frog sampled across the three sampled time points. Lines represent samples taken from the same individual frog.
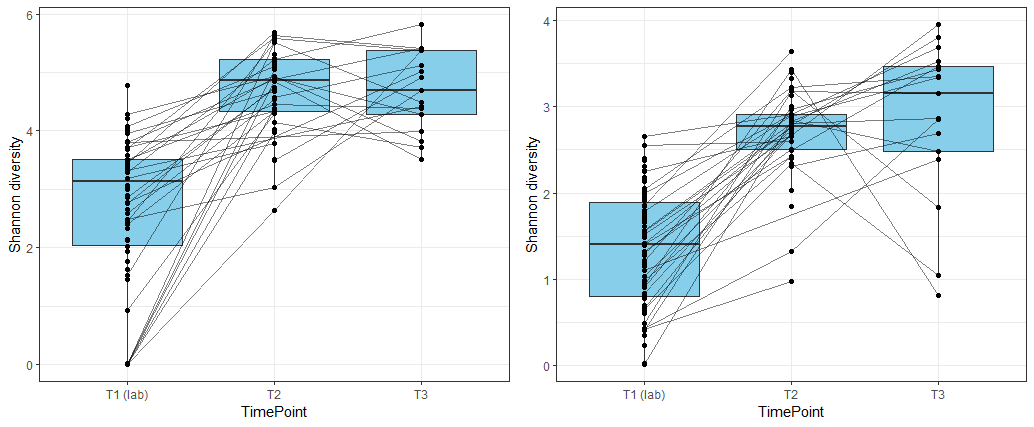


Fig. S4) Residual associations between the 50 most common bacterial and fungal genera found on the skin of the Southern Corroboree frog. Associations are extracted from a null GLLVM model, and indicates strong correlations between genera when not accounting for shared responses to temporal and spatial factors.


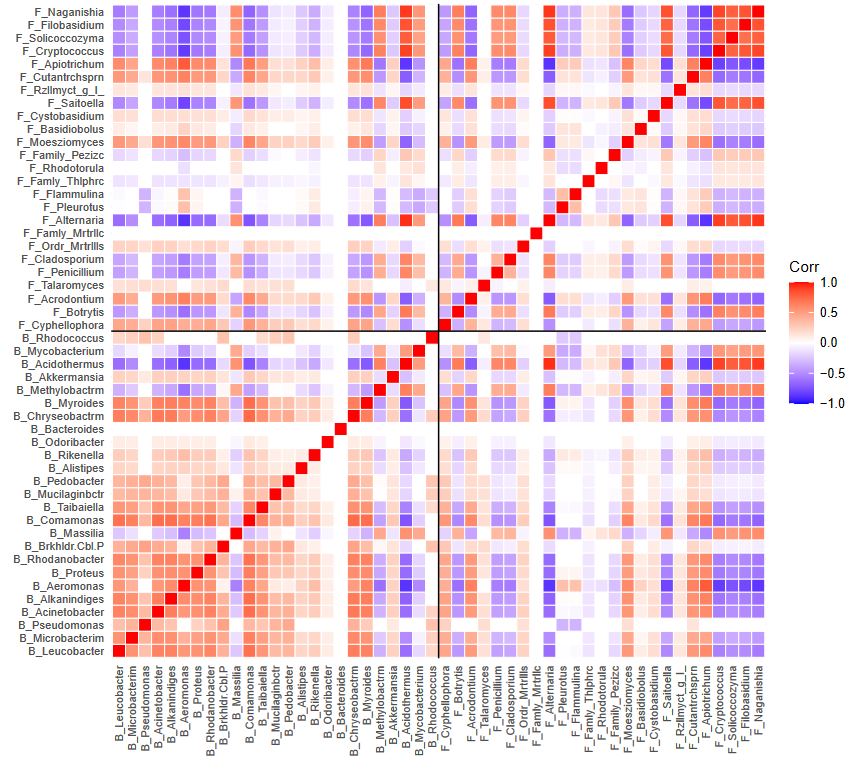


Fig. S5) The same residual correlation network shown in Figure 5b, but with genus names indicated. Bacteria are coloured yellow and fungi are coloured blue. Negative associations are represented by blue, and positive association are represented by red.


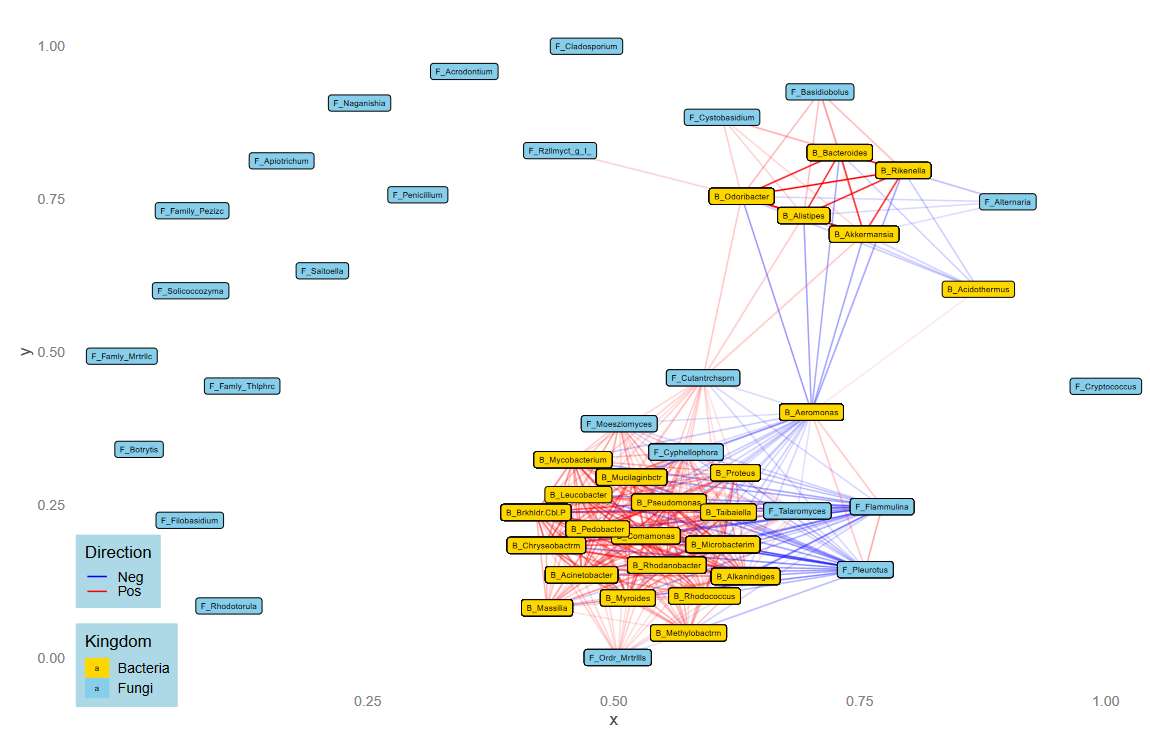


**Supplementary tables**

Table S1) Composition of experimental diets fed to captive P. corroboree metamorphs prior to field release.

| Treatment | β-carotene mass (g) | β-carotene concentration per g of feed (mg g-1) | Cellulose mass (g) | Calcium mass (g) | Total supplement mass (g) |
| --- | --- | --- | --- | --- | --- |
| C0 | 0 | 0 | 0.045 | 0.25 | 0.295 |
| C1 | 0.015 | 1 | 0.03 | 0.25 | 0.295 |
| C2 | 0.03 | 2 | 0.015 | 0.25 | 0.295 |
| C3 | 0.045 | 3 | 0 | 0.25 | 0.295 |

|  | **Bacteria** | | | | |  | **Fungi** | | | | |
| --- | --- | --- | --- | --- | --- | --- | --- | --- | --- | --- | --- |
| **a) Pre-release** | Term | Est | Std error | Statistic | P val |  | Term | Est | Std error | Statistic | P val |
|  | **Intercept (C0)** | **41.46** | **13.27** | **3.12** | **<0.001** |  | **Intercept (C0)** | **9.12** | **1.46** | **6.26** | **<0.001** |
|  | Treatment (C1) | 8.24 | 15.77 | 0.52 | 0.60 |  | Treatment (C1) | 2.37 | 2.05 | 1.16 | 0.25 |
|  | Treatment (C2) | 4.51 | 16.24 | 0.28 | 0.78 |  | Treatment (C2) | 2.11 | 2.13 | 0.99 | 0.32 |
|  | Treatment (C3) | 10.74 | 15.63 | 0.69 | 0.49 |  | Treatment (C3) | 0.77 | 2.16 | 0.36 | 0.72 |
|  | **Seq. depth** | **0.00** | **0.00** | **5.58** | **<0.001** |  | Seq. depth | 0.00 | 0.00 | 1.14 | 0.25 |
|  |  |  |  |  |  |  |  |  |  |  |  |
|  | Term | Est | Std error | Statistic | P val |  | Term | Est | Std error | Statistic | P val |
| **b) Post-release** | **Intercept (C0)** | **129.43** | **26.80** | **4.83** | **<0.001** |  | **Intercept (C0)** | **23.53** | **4.78** | **4.92** | **<0.001** |
|  | Treatment (C1) | 34.20 | 32.69 | 1.05 | 0.30 |  | Treatment (C1) | 5.92 | 6.74 | 0.88 | 0.38 |
|  | Treatment (C2) | 16.40 | 32.11 | 0.51 | 0.61 |  | Treatment (C2) | 8.51 | 6.64 | 1.28 | 0.20 |
|  | Treatment (C3) | -14.70 | 33.31 | -0.44 | 0.66 |  | Treatment (C3) | 0.80 | 7.11 | 0.11 | 0.91 |
|  | Seq. depth | 0.00 | 0.00 | 0.48 | 0.63 |  | Seq. depth | 0.00 | 0.00 | 0.65 | 0.51 |

Table S2) General linear models predicting the effect of ꞵ-carotene treatment on ASV richness (alpha diversity) of skin bacterial and fungal communities on samples collected a) prior to release and b) post-release of Southern Corroboree frogs. Significant associations are highlighted in bold.
